# DHA Supplementation during Pregnancy and Lactation Affects Infants' Cellular but Not Humoral Immune Response

**DOI:** 10.1155/2011/493925

**Published:** 2011-09-18

**Authors:** Esther Granot, Einat Jakobovich, Ruth Rabinowitz, Paloma Levy, Michael Schlesinger

**Affiliations:** ^1^Department of Pediatrics, Kaplan Medical Center, P.O. Box 1, Rehovot, Israel; ^2^Hadassah Medical School, Hebrew University, Jerusalem, Israel; ^3^Department of Developmental Biology and Cancer Research, Hadassah Medical School, Hebrew University, Jerusalem, Israel

## Abstract

*Background*. It is currently recommended that diet of pregnant mothers contain 200–300 mg DHA/day. *Aim*. To determine whether DHA supplementation during pregnancy and lactation affects infants' immune response. *Methods*. 60 women in ≥3rd pregnancy studied; 30 randomly assigned to receive DHA 400 mg/day from 12th week gestation until 4 months postpartum. From breast-fed infants, blood obtained for anti-HBs antibodies, immunoglobulins, lymphocyte subset phenotyping, and intracellular cytokine production. *Results*. CD4+ lymphocytes did not differ between groups, but CD4CD45RA/CD4 (naïve cells) significantly higher in infants in DHA+ group. Proportion of CD4 and CD8 cells producing IFN_*γ*_ significantly lower in DHA+ group, with no differences in proportion of IL4-producing cells. Immunoglobulins and anti-HBs levels did not differ between groups. *Conclusions*. In infants of mothers receiving DHA supplementation, a higher percentage of CD4 naïve cells and decreased CD4 and CD8 IFN_*γ*_ production is compatible with attenuation of a proinflammatory response.

## 1. Introduction

Breast feeding is believed to confer protection against infections during infancy. Breast-fed infants have an enhanced local humoral immune response, resulting in a lower prevalence of gastrointestinal and respiratory tract infections than in formula-fed infants [1, 2]. Exclusive breast feeding for the first few months has also been suggested to be protective against the development of atopic disease [3]. Immunoglobulins, lymphocytes, proteins like lactoferrin, and lysozyme, which are present in breast milk, play a specific immunologic role. Another component, which may be of pivotal importance to maturation and function of the immune system, is the fatty acid pattern of the milk. Human milk contains long chain polyunsaturated fatty acids (LCPUFA) (20–22 carbons) of both w-3 and w-6 classes which constitute ~2% of total fatty acids and which are undetectable in unsupplemented formulas prepared from vegetable oils. RBC membrane phospholipids of breast-fed infants have a significantly higher percentage of w-3 fatty acids (FA) and specifically a twofold higher level of docosahexanoic acid (DHA) (C22:6 w-3) than infants fed by nonsupplemented formula, with similar membrane levels of arachidonic acid (AA) (C 20:4 w-6) [4, 5]. The balanced ratio of arachidonic acid-derived eicosanoids and those derived from w3 fatty acids has been suggested to play a crucial role in immune modulation [6–8].

In infants who are breast fed, supply of these fatty acids is dependent on the maternal diet [9]. The Western diet contains large amounts of w-6 FA but is relatively low in w-3 FA. Maternal DHA status depends mainly on intake of DHA itself as conversion of alpha-linolenic acid (C18:3 w-3), present in nuts and other vegetable oils, into DHA is low. Levels of maternal DHA decline during pregnancy and decrease even further when the lactation period is extended [10]. Maternal DHA levels decline with multiple pregnancies; levels have been shown to be significantly lower in multiparous compared with primiparous mothers and when pregnancies are closely spaced. Furthermore, levels of DHA in breast milk correlate with DHA blood levels [11]. 

The high demand for DHA has prompted current recommendations that during pregnancy and lactation the average dietary intake of DHA should be 200–300 mg per day [12, 13]. Indeed, recent studies have established that the diet of Western pregnant and lactating women contains only 20%–60% of the w-3 FA recommended daily intake. In 90% of the women surveyed, DHA intake was far below the recommended requirement [14, 15].

Previous studies relating to the immune system in infants have compared infants fed human milk with infants fed either LCPUFA-supplemented or nonsupplemented formulas [16, 17]. In pregnant women receiving a DHA + EPA preparation, from the 22nd gestational week, maternal blood mRNA levels of Th1 cytokines (IFN*γ* and IL1) were decreased, whereas, in cord blood, levels of Th2 cytokines (IL4 and IL13) were decreased [18]. No studies have examined the effect of maternal DHA supplementation during pregnancy and lactation on the infants' immune system.

We therefore questioned whether DHA supplementation during pregnancy and lactation, in a population with a high percentage of multiparous mothers with closely spaced pregnancies, may affect the humoral and cellular immune response in infants solely breast-fed up to the age of 4 months.

## 2. Subjects and Methods

### 2.1. Subjects

The mothers of the babies studied belong to an ultraorthodox religious community; 60 pregnant women, age 20–35 years, in their ≥3rd pregnancy. 30 women were randomly assigned to receive DHA 400 mg/day (each softgel capsule containing 100 mg DHA, produced by Martek Biosciences Corporation, Solgar, Leonia, NJ, USA). DHA supplementation was taken daily from the 12th week of gestation and continued until the end of the 4th postpartum month. Mothers who were not assigned to the DHA supplementation group declined intake of placebo capsules on religious grounds, as the gelatin capsules are not “kosher”, but since the DHA was regarded by the religious leaders of the community as a medication, intake of DHA capsules was permitted. In the DHA-supplementation group, 18 mothers were in their 3rd-4th pregnancy, 11 were in their 5th–7th pregnancy, and 1 was in her 8th. In the control group, 17 mothers were in their 3rd-4th pregnancy, 11 in the 5th–7th, and 2 in the ≥8th pregnancy. 

A nutritional intake questionnaire, with an emphasis on type of cooking oil used, amount and type of fish, and/or nuts consumed, was filled out by all mothers. 

Infants were exclusively breast-fed for 4 months. At age of 4 months, blood was drawn (maximum 5 mL of which 2.5 mL in EDTA and 2.5 mL in heparin) for complete blood counts (CBC), anti-HBs antibody titers, immunoglobulin levels, and lymphocyte studies.

### 2.2. Methods

#### 2.2.1. Anti-HBs Antibodies

Quantitatively determined using a microparticle enzyme immunoassay 9AxSYMAVSAB, Abbot Diagnostics, Wiesbaden, Germany.

#### 2.2.2. Immunoglobulin Levels

Quantitative determination of immunoglobulins (IgA, IgM, IgG) was performed by means of immunonephelometry using BN ProSpec Systems, Siemens Healthcare Diagnostics, Marburg, Germany.

#### 2.2.3. Lymphocyte Studies

**Monoclonal Antibodies**

The following monoclonal antibodies were obtained from Immunoquality Products, Groningen, the Netherlands: anti-CD8, anti-CD45RO, anti-CD45RA, and anti-CD25. Anti-CD4 and anti-CD6 mAb were purchased from eBioscience, Inc., San Diego, Calif.

MAbs for the detection of intracytoplasmic cytokines were obtained from BD Biosciences (San Jose, Calif). Fastimmune PE-labeled antihuman IL4 was used for the detection of IL4, while Fastimmune FITC-labeled antihuman interferon-*γ* (IFN*γ*) was used for the detection of IFN*γ*.

**Immunofluorescent Staining of Peripheral Blood Lymphocyte Subsets**

The following peripheral blood lymphocyte subsets determined by cell surface staining using a fluorescent activated cell sorter: CD4, CD4CD45RA (“naïve” T helper cells), CD4CD45RO (“memory”/activated helper cells), CD8, CD8CD45RA (“naïve” T suppressor cells), CD8CD45R0 (“memory”/activated suppressor cells), and CD16 (NK cells). Whole blood cells, in heparin, were washed twice in PBS and suspended in PBS containing 0.1% BSA and 0.1% NaN_3_. Suspensions at a volume of 100 *μ*L, were introduced into each test tube and stained with mAbs. The cells were incubated with either FITC- or PE-conjugated mAbs for 30 min at 4°C. The mixture of cells and antibody was incubated for 30 min at 4°C. After incubation the red blood cells were lysed with FACS lysing solution (Becton Dickinson). After two incubations of the blood with NH_4_CL for 10 minutes at room temperature, the suspension was centrifuged, the pellet washed twice with PBS, and cells resuspended in 1% paraformaldehyde and stored at 4°C until they were analyzed by flow cytometry [19].

#### 2.2.4. Cell Activation for Cytokine Production

0.5 mL whole blood, in heparin, was introduced into disposable polystyrene tubes. To 0.5 mL blood, 0.5 mL RPMI-1640 medium and 2 mM L-glutamine were added. The tissue culture medium components were purchased from Biological Industries, Beit Haemek, Israel. For activation cells were exposed to mitogens, 25 ng PMA and 1.0 *μ*g ionomycin. 10 *μ*g brefeldin A (BFA) were added to each tube; BFA inhibits intracellular transport and prevents secretion of the intracellular cytokines. The suspensions were incubated at 37°C in a 5% CO_2_ humidified atmosphere for a 4-hour incubation.

#### 2.2.5. Intracytoplasmic Immunofluorescent Staining of Cytokines in Activated Cells

For intracytoplasmic immunofluorescent staining, the cells were resuspended in 500 *μ*L FACS permeabilizing solution, obtained from Becton Dickinson, and were left for 8–10 minutes at room temperature. The cells were washed twice in PBS containing 0.5% BSA and 0.1% NaN_3_. 20 *μ*L of fluorescent conjugated mAbs were added to stain intracytoplasmic cytokines. IgG preparation of suitable isotypes was used as negative controls [20]. The cells were incubated for 30 minutes at room temperature, washed and fixed in a solution of 1% paraformaldehyde in PBS, and stored at 4°C until they were analyzed by flow cytometry. Acquisition and analysis were carried out in a FACScan flow cytometer (Becton Dickinson) using the FACScan software program. For each test, 5000–10000 cells were collected and acquired. Because of the small amounts of blood that could be obtained, these assays were performed in only 21 infants, in each group. 

The study was approved by the Ethics Committee (IRB) of the Kaplan Medical Center and the Review Board of the Ministry of Health.

### 2.3. Statistical Analysis

Statistical analysis for evaluation of significant differences was performed with the use of Student's *t* test for unpaired data with InStat (GraphPad Software Inc. San Diego, CA). Results were presented as mean ± SEM.

## 3. Results

### 3.1. Demographic Characteristics

Demographic characteristics of the mothers and infants studied are summarized in Table 1. Mothers' age and parity did not differ between mothers receiving DHA supplementation and the nonsupplemented group. Infants' birth weight, gestational length, and number of infectious episodes documented until performance of immunology studies, at age 4 months did not differ between groups.

### 3.2. Anti-HBs Antibodies

117.9 ± 34.3 IU/mL (mean ± SEM) in infants of DHA-supplemented mothers versus 111.9 ± 56.1 IU/mL in the nonsupplemented group (*P* = n.s).

### 3.3. Immunoglobulins

IgA levels were 33.9 ± 4.22 mg% (mean ± SEM) in the DHA group versus 54.5 ± 15.6 mg% in controls (*P* = n.s). IgM levels were 99.7 ± 15.0 mg% in the DHA group versus 91.8 ± 20.8 mg% in controls (*P* = n.s). IgG levels were 593.6 ± 70.3 mg% in the DHA group versus 745.7 ± 162.7 mg% in controls (*P* = n.s).

### 3.4. Total Lymphocyte Count

Total lymphocyte count did not differ between the two groups of babies; 6598 ± 450 /mm^3^ (mean ± SEM) in the babies of supplemented mothers versus 6900 ± 650 /mm^3^ (mean ± SEM) in controls (*P* = n.s).

### 3.5. Lymphocyte Subsets

In the babies of w3-supplemented mothers, the percentage of CD4+ lymphocytes did not differ significantly from that of the control group (46.9 ± 1.61 versus 41.0 ± 2.75, *P* = n.s), but CD8+ lymphocytes were significantly lower (17.7 ± 0.98 versus 21.25 ± 0.09, *P* < 0.05) (Table 2). Among CD4+ cells, the proportion of the CD4+CD45RA+ subset, representing naïve helper cells, was significantly higher in the infants of w3-supplemented mothers as compared with the control group. The proportion of CD45RA+CD4+ cells constituted 87.23 ± 0.89 of total CD4 population in the supplemented group versus 79.8 ± 2.95 in controls (*P* < 0.05). The percentage of memory CD4 cells, CD4+CD45RO+/CD4, did not differ between the two groups (15.79 ± 1.77 versus 12.80 ± 2.01 (*P* = n.s)). Of CD8+ cytotoxic suppressor cells, although the percentage of CD8 cells was lower among infants of mothers receiving DHA supplementation, the CD8+CD45RO+/CD8 fraction, representing memory cells, was higher in the DHA-supplemented group; 34.9 ± 5.9 versus 16.2 ± 5.9 (*P* < 0.05). The sum of the two CD8 fractions, naïve CD8+ cells (CD8+CD45RA+), and activated CD8+ cells (CD8+CD45RO+) is higher than 100%, denoting that a proportion of the cells has stained positive for both markers, and is a phenomenon observed during the transition from a naïve to an activated state. The proportion of CD16+ lymphocytes did not differ significantly between the two groups (14.92 ± 9.08 versus 14.58 ± 5.51, in DHA-supplemented and control groups, resp.).

### 3.6. Cytokine Production

The proportion of CD4+ and of CD8+ producing IFN*γ* was significantly lower in the DHA-supplemented group (Table 3). The proportion of IFN*γ* producing CD4+ cells was 0.99 ± 0.18% among the DHA-supplemented group versus 2.68 ± 0.69 among controls (*P* = 0.01). Similarly among CD8+ cells, the proportion of IFN*γ* producing cells was 0.62 ± 0.13 versus 1.84 ± 0.43, respectively, (*P* = 0.01). IL4 production in CD4+ and CD8+ cells was lower in the DHA-supplemented group, but differences did not reach statistical significance (Table 3).

## 4. Discussion

The peripheral blood lymphocyte profile of breast-fed infants of mothers who have received DHA supplementation during pregnancy and lactation differs from that of breast-fed infants whose mothers' diet was not supplemented with DHA. 

Infants in the DHA-supplemented group had a similar number of CD4 cells as the nonsupplemented group, but the number of CD8 cells was significantly reduced so that the CD4/CD8 ratio was 2.6 among lymphocytes of the DHA-supplemented infants as compared with 1.9 in the nonsupplemented group. Of CD4 cells the fraction of CD45RA+ cells, representing naïve helper cells, was significantly higher in the DHA-supplemented group than in the nonsupplemented group. Although total population of CD8 cells was lower in DHA-supplemented infants, the proportion of CD8+CD45RO+ activated cells was significantly higher, constituting 35% of CD8 cells as compared with 16% in the nonsupplemented group. In both CD4 and CD8 cells, the production of IFN*γ* was found to be markedly lower in lymphocytes of the infants in the DHA-supplemented group. 

Infants of both groups resided in the same neighborhood and were likely exposed to similar environmental antigens and infectious hazards. The differences in the level of lymphocyte subsets between the two groups of babies can therefore not be attributed to a different degree of bacterial or viral exposure. 

Field et al. [16] have previously studied the immune consequences of adding arachidonic acid (ARA) and DHA to preterm formula-fed infants as compared with human milk-fed preterm infants. At 42 days of age, the lymphocyte subset population in infants receiving an LCPUFA-fortified formula showed an increase in the proportion of CD4CD45RO+ cells and CD8CD45RO+ cells over that present in peripheral blood of formula-fed infants, a lymphocyte subset pattern more consistent with that observed in infants fed human milk. Activation of IL2, a major proinflammatory cytokine, measured indirectly based on the amount of SIL-2R produced by peripheral mononuclear cells, was noted to be lower in LCP-fed infants with levels comparable to those in human-milk-fed infants. This observed decrease in IL2 activation is in agreement with other studies both in vitro and in vivo (animals and adults) that have demonstrated that LCPUFA reduce IL2 production [21]. Production of TNF*α*, another proinflammatory cytokine, from mononuclear cells, has also been shown to decrease in adults receiving LCPUFA [22]. A recent study has demonstrated decreased release of IFN*γ*-induced protein from airway epithelial cells infected with rhinovirus, when incubated with DHA [23].

 In a study conducted in children aged 5–7 years consuming a diet with low DHA intake, adding ARA and DHA (14–21 mg/day) for a 7-month period did not lead to any changes in relative proportion of CD4 and CD8 cells, in non-stimulated whole blood.

Furthermore, IFN*γ* production by peripheral blood mononuclear cells, following exposure to various stimulants/mitogens, did not differ in the LCPUFA-supplemented group [17]. Another study, in older children aged 8–12 years, reached different conclusions regarding the effect of w-3 FA on cytokine production. In children receiving 300 mg fish oil (+700 mg canola oil) per day for 12 weeks, peripheral blood mononuclear cells incubated in absence or presence of lipopolysaccharide stimulation exhibited a nonspecific enhancement of both proinflammatory and anti-inflammatory down regulatory cytokine production [24]. Notably, while some in vitro and in vivo studies, in animal models and adults, have also noted that LCPUFA induce an increase of TNF, IL1, and IL2 production, others have failed to show any effect of different doses of fish oil on production of these cytokines [7, 25–27]. 

Most studies have assayed the level of cytokines present in supernatants of isolated peripheral blood mononuclear cells or lymphocyte cultures. In our study, utilization of a technique for the intracellular staining of cytokines enabled us to determine the selective origin of cytokines, whether from CD4 or CD8 cells. 

DHA supplementation did not exert an effect on the humoral immune response. Infants in both groups did not differ in levels of immunoglobulins of IgG, IgM, and IgA class.

Specific antibody response as reflected by measuring the titer of antibodies to HBsAg after 2 doses of vaccine (at 2 days and at 1 month of age) was similar in both groups. 

All infants in our study were exclusively breast fed for the first 4 months of age. As for the diet of the mothers taking part in the study, they all ate at least 2 servings of fish per week, cooked with canola oil, which is rich in w-3 FA, and added olive oil as salad dressing. A recent consensus statement regarding dietary fat intake for pregnant and lactating women has concluded that “ the desired average intake of at least 200 mg DHA/day can be reached with the consumption of one to two portions of sea fish per week” [13]. Thus, their dietary intake of DHA may be considered as adequate.

Our study is unique in that it questioned whether additional DHA supplementation to breast-feeding mothers who are consuming a diet supposedly sufficient in DHA content would affect the immune response of their infants. 

DHA supplementation during pregnancy and lactation did not result in changes in the infants' humoral immune response, as assessed by levels of anti-HBs antibodies and total immunoglobulins, but did affect lymphocyte subset profile and cytokine production.

A lymphocyte profile with a higher percentage of CD4 naïve cells and decreased IFN*γ* production in both CD4 and CD8 cells is likely compatible with attenuation of a proinflammatory response.

Local expression of proinflammatory cytokines, particularly IFN*γ* and TNF, has been shown, in animal models, to worsen damage as incurred, for example, during ischemic strokes [28]. Dampening of the proinflammatory response may thus serve to limit tissue damage during infectious and noninfectious inflammatory situations. Yet it is noteworthy that although interferon-*γ* is considered to be an important proinflammatory cytokine produced by Th1 lymphocytes and by natural killer cells, mediating both innate and adaptive immune response, recent observations indicate that IFN*γ* may exert suppression of the immune response and may play a role in the induction of tolerance.

IFN*γ* has been shown to modulate T-cell activation by reducing T-cell proliferation and survival and by increasing the number of regulatory T cells (Foxp3+ Treg cells) [29, 30]. IFN*γ* also stimulates the expression of inhibitory B7 family molecules, which in turn may abate the production of Th1 T cells [31]. Thus, the immunological consequences of the striking decrease in IFN*γ* production in both CD4 and CD8 cells cannot be simply predicted. As for the observed higher percentage of cytotoxic activated CD8 cells, these may play a facilitatory role in encountering of viral infections. 

The clinical implications of the changes observed are as yet speculative, and whether supplementation of DHA to breast-feeding mothers confers an advantage to their infants remains to be elucidated.

##  Authors' Contribution

E. Granot, Principal Investigator, helped in initiating and designing of project, analysis of data, and writing of manuscript and took the responsibility for its final content. E. Jakobovich, Study Coordinator, helped in the clinical followup of mothers and infants and data collection. R. Rabinowitz and P. Levy performed lymphocyte studies and contributed to analysis of data. M. Schlesinger is director of the laboratory in which lymphocyte studies were performed and is responsible for planning of lymphocyte studies and analysis of the data. All authors read and approved the final paper.

##  Conflict of Interests

The authors have no conflict of interests to report.

## Figures and Tables

**Table 1:** Characteristics of mothers and infants studied.

	DHA-supplemented group	Control group
Mothers		
Age (y)	27.7 ± 4.2	27.6 ± 4.6
Pregnancy (no.)		
3rd-4th	18	17
5th–7th	11	11
≥8th	1	2

Infants		
Birth weight (kg)	3.33 ± 0.4	3.30 ± 0.5
Gestational age (weeks)	40.0 ± 1.6	39.9 ± 0.9
Infectious episodes (no.)	2 (fever/URI)	1 (pertussis)
Birth-4 months		
Nutrition	Solely breast fed	Solely breast fed

Data are mean ± SD, *P* = n.s.

**Table 2:** The effect of maternal DHA supplementation on phenotype of infants' peripheral blood lymphocytes.

	DHA-supplemented group	Control group	
CD4+ cells	46.93 ± 1.61	41.02 ± 2.75	*P* = n.s
CD8+ cells	17.67 ± 0.98	21.25 ± 0.99	*P* = 0.01
CD45RA+ CD4+ CD4+	87.23 ± 0.89	79.86 ± 2.95	*P* < 0.05
CD45RO+ CD4+ CD4+	15.79** ± **1.77	12.80 ± 2.01	*P* = n.s
CD45RA+ CD8+ CD8+	94.02 ± 0.85	90.08 ± 1.45	*P* < 0.05
CD45RO+ CD8+ CD8+	35.51 ± 6.20	16.80 ± 5.42	*P* < 0.05

Data are mean ± SEM, expressed as % of peripheral blood lymphocytes.

**Table 3:** The effect of maternal DHA supplementation on cytokine production by infants' peripheral blood lymphocytes.

	DHA-supplemented group	Control group	
CD4+ IL4+	0.69 ± 0.12	1.24 ± 0.56	*P* = n.s
CD8+ IL4+	0.26 ± 0.07	0.68 ± 0.31	*P* = n.s
CD4+ IFN*γ*+	0.99 ± 0.18	2.68 ± 0.61	*P* = 0.01
CD8+ IFN*γ*+	0.62 ± 0.13	1.84 ± 0.43	*P* = 0.01

Data are mean ± SEM, expressed as % of peripheral blood lymphocytes.
